# Service Learning Experience on Health Habits in High School Students Conducted by Nursing Students: A Qualitative Design

**DOI:** 10.3390/nursrep12040078

**Published:** 2022-10-29

**Authors:** Reig-Garcia Glòria, Cámara-Liebana David, Maria Carme Malagón-Aguilera, Belén Pérez-Jiménez, Susana Mantas-Jiménez, Marta Roqueta-Vall-llosera

**Affiliations:** 1 Department of Nursing, Faculty of Nursing, University of Girona, 17003 Girona, Spain; 2 Health and Health Care Research Group, University of Girona, 17003 Girona, Spain; 3 Quality of Life Research Institute, University of Girona, 17003 Girona, Spain; 4 Health Care Area of Salt, Catalan Health Institute, 17190 Salt, Spain; 5 Health, Gender and Aging Research Group, University of Girona, 17003 Girona, Spain

**Keywords:** service learning methodology, nursing students, high school, teenagerhood, health habits

## Abstract

Service learning is a teaching methodology that combines learning and service to the community in the same well-articulated project, where the participants are trained to improve and work on the real needs of the environment. This paper aimed to explore learning about healthy habits and standards of nursing professional practice through a service learning activity between nursing students and high school students. Data of participants (N = 127 high school students and N = 12 nursing students) were collected by the high school students’ healthy habits mind map and with the help of the nursing students’ reflective journals. A generic qualitative design using content analysis was performed. After the activity, high school students identified which habits they should improve, such as diet, physical activity, resting time, and emotional health. By performing the activity, nursing students increased their knowledge about health habits, as well as their communication skills, confidence in public speaking, and awareness about community nurses’ tasks. Service learning activity on health habits conducted by nursing students in a high school has a positive effect on the knowledge of healthy habits for both participants, nursing and high school students. Participating in an activity of service learning improves communication skills among first-year nursing students and narrows the gap between university theory and nursing practice.

## 1. Introduction

Health literacy is considered by the World Health Organization (WHO) to be one of the pillars of health promotion and a critical determinant of health for people’s empowerment. In 2016, educational settings were highlighted during the Ninth Global Conference on Health Promotion as important settings for investing in the development of health literacy for young people through life skills-based school curricula [1,2]. In addition, one of the targets of the Sustainable Development Goals by 2030 is to provide students with all the knowledge and skills necessary for their own sustainable development [3]. Utilizing school health education to promote health literacy can be challenging, but it is a basic prerequisite for students’ empowerment and enables them to adopt healthy lifestyles over their lifetimes [1,2].

Adolescence is defined by the WHO as the period of life between 10 and 19 years of age [1]. This is particularly a stage of life that transitions from childhood to adulthood in which values and attitudes are built, such as lifestyles, impacting current and future health [1,4]. Therefore, investing in teenagers’ health can bring about substantial health, economic, and social benefits [1]. Recent research highlighted some prevalence of non-communicable diseases among adolescents [5,6,7,8]. There are four main modifiable risk behaviors that contribute to most of the global burden of non-communicable diseases, namely: unhealthy diet, physical inactivity, and tobacco and alcohol use [4,9]. Because high school is a natural space for learning, education, and comprehensive training of the person, it could be a key area for promoting health.

Therefore, since 2004, the Department of Education and the Department of Health in Catalonia (Spain) proposed a series of actions involved in the Health and School Program, which are aimed at detecting the most relevant health problems affecting teenagers in order to offer health promotion activities [10].

On the one hand, depending on the number of high school centers existing in the territorial scope of the community area, the number of health professionals participating in the program was variable, which was preferably community nurses. On the other hand, students in the first and second year of secondary education were the other group of actors involved in the Health and School Program. Each reference nurse of the program worked in the high school, giving health promotion and prevention sessions in groups, as well as offering a space where high school students could access spontaneously and freely to consult health-related topics concerning them [10].

Service learning as a teaching methodology integrates the learning of content, skills and values, and service to the community. Therefore, it combines learning and service to the community in the same well-articulated project in which the participants are trained to work on the real needs of the environment in order to improve it. Thus, it is an educational proposal with social utility [11]. Service learning is inspired by an active and reflective pedagogy that is based on: experience, active participation, reflection, interdisciplinarity, problem solving, and cooperation. Thus, service learning requires a network that promotes and guarantees the coordination of formal educational institutions with social organizations to intervene in reality and influence the development of the service [11]. Service learning projects can be developed in different areas, influencing public policies and improving the quality of life of its inhabitants in order to develop basic skills in health promotion, such as community participation projects and health promotion projects [12,13]. Boswell [14] identified service learning as a strategy to overcome the gap between theory and practice. Therefore, nursing curricula should adopt service learning methodologies as an experiential learning pedagogy to provide opportunities for students to engage with their community and increase their knowledge. Moreover, as service learning takes place in a real-life situation, this provides essential knowledge and experience needed for caring for diverse community backgrounds as future registered nurses [15,16,17].

In Catalonia (Spain), nursing degrees are carried out in 4 academic years and combine classes at the university and practicum placement [18]. *Healthy Person Care throughout the Life Cycle* is a subject in the first year of the degree [18] and is known to be a relevant topic. It encompasses knowledge of the person throughout their lifecycle, from birth to the final stages of life, as well as their needs and preferences [19,20]. *Healthy Person Care throughout the Life Cycle* includes content related to health determinants and inequalities during the different stages, such as childhood, adolescence, adulthood, and senectitude. Healthy habits are studied in the adulthood block, where nursing students are asked to prepare a real session on this topic to be developed later in a real environment under the umbrella of the service learning methodology [18]. The aim of the present study was to explore learning about healthy habits and standards of nursing professional practice through a service learning activity between nursing students and high school students.

## 2. Methods

### 2.1. Design

This was a qualitative study performed during the academic year 2021–2022 that gathered data on service learning methodology in nursing studies. A generic qualitative design [21] based on a constructivist naturalistic approach was adopted. A qualitative methodology offers the possibility of understanding the complexity of a phenomenon from the differing points of view of informants [22]. Generic studies offer an opportunity for researchers to play with boundaries, use the tools provided by established methodologies, and develop research designs that fit their epistemological stance, discipline, and particular research questions [23].

### 2.2. Sample

The study population consisted of 12 1st-year students of nursing degree at the Faculty of Nursing, part of Universidad de Girona(Spain), and 124 1st-year students of secondary education at a high school in the same region (Spain). 

The recruitment of the 12 nursing students was based on the qualification of the activity on healthy habits performed in the subject of *Healthy Person Care throughout the Life Cycle.* In order to develop this activity, we only needed high school students from one high school. Therefore, the high school where the activity was carried out was randomly selected by a nurse from the Health and School program among more than 70 centers. 

The inclusion criteria for the nursing students were: being a 1st-year student of the nursing degree, receiving the best qualification in the activity related to healthy habits in the subject of *Healthy Person Care throughout the Life Cycle,* and being willing to attend a session on healthy habits to a group of high school students. Inclusion criteria for the teenage population were: being a student in the 1rst-year of high school education in the selected center and wanting to participate in the activity. 

Nursing students were invited to participate in the study by the lecturers of the subject, who explained the objectives and the procedure of the study. Moreover, the high school was selected randomly and invited to the study by the Health and School program territorial reference nurse. First-year students of the high school were informed about the activity to be carried out and invited to participate by the nurse of the Health and School program of their high school. As it is an activity developed through the Health and School program [10], the informed consent of the parents of high school students was already obtained when the parents received the information about the program and which activities are included in it. In addition, before starting the sessions on health habits carried out by nursing students, the nurse of the Health and School Program explained the activity to high school students and also asked again for oral consent to participate in the proposed activity. 

### 2.3. Procedure and Instruments

Students enrolled in the subject of *Health Person Care throughout the Life Cycle* (1st year of the Nursing Degree) during the second semester were proposed to participate in the activity: Learning Healthy Habits through the Service Learning Methodology. The activity consisted of preparing and performing, in groups of 3 or 4 students, a practical session about healthy habits among teenagers. The theoretical content of health habits needed to be prepared using different sources before the session, such as books, articles, official health channels, etc. The students who obtained the best qualification in this activity and who voluntarily wanted to participate in the service learning were invited to participate in the study. 

Once the nursing students were selected, a meeting between them, the lecturers of the subject, and the Health and School program reference nurse was conducted to review and adapt the contents and dynamics of the health habits session. 

During May 2022, 12 nursing students, 2 to 3 for each group, gave the same session on healthy habits to the 1st year students in 5 different high school classes. These sessions were held in the space and time that the high school reserved for activities related to the Health and School program. Therefore, during the sessions, the students were accompanied by the reference nurse of the Health and School program, who had an observing and supporting role. Each session was carried out in the format of a theoretical and practical workshop lasting approximately 60 min. The practical workshop was developed through a dynamic group where high school students identified areas of healthy habits to be improved and shared with the rest of the class group using a mind map. The mind map tool was designed to allow high school students to identify, through a notes wall, the health habits they thought needed to be improved. After the session, this healthy habits mind map was collected for analysis. Nursing students recorded their experience using a reflective journal, which explored areas of interest, such as global satisfaction with the activity, perception of learning about health behaviors, and other learnings regarding standards of nursing professional practice. In addition to examining the nursing students’ experience related to the activity, we reviewed the reflective journals using the Gibbs Reflective Cycle, which encompasses 6 stages: description of the experience, feelings and thoughts about the experience, evaluation of the experience, analysis to make sense of the situation, conclusions about the learning, and action plan [24].

### 2.4. Ethical Considerations

This study was approved by the Faculty of Nursing management, the High School administration, and the Community Health Care management of the region. 

All the participants were informed about the objective of the study. Informed consent from the nursing students was obtained. Regarding informed consent of high school students, being an activity developed through the Health and School program, the parents of high school students had already consented to their participation in the activity. In addition, before starting the session on health habits carried out by nursing students, the nurse of the Health and School Program explained the activity to high school students and also asked for oral consent to participate in the proposed activity.

Anonymity was maintained at all times. Information collected by the healthy habits mind map and the reflective journals did not contain any personal information that could identify participants. The principles defined in the Declaration of Helsinki were followed. 

### 2.5. Data Analysis 

Both the data obtained from the healthy habits mind map and the reflective journals were analyzed by two different researchers using content analysis. These two authors analyzed all the data independently. Afterward, all the themes were discussed and clarified until a consensus was reached [25]. 

Krippendorff [26] defined content analysis as “a research technique for making replicable and valid inferences from texts (or other meaningful matter) to the contexts of their use”. The process followed in conducting qualitative content analysis comprises four stages: decontextualization, recontextualization, categorization, and compilation [27]. The Standards for Reporting Qualitative Research (SRQR) checklist was used to ensure the rigor of the research [28]

## 3. Results

### 3.1. Results Related to the High School Students

First, a health mind map activity was conducted by 124 of the 1st-year students of secondary education at a high school. Of these, 57% identified as female, and 43% identified as male. The average age of the participants was 14 years old (*SD* = 0.7).

The high school students participated actively in the theoretical session, and afterward, they were able to identify which of their habits were healthy or unhealthy. 

According to the content analysis, high school students felt that it was necessary to modify and incorporate improvements into the following health habits: (a) diet, (b) physical activity, (c) resting time, and (d) emotional health. Regarding tobacco consumption, there was no intention to change their habits 

Regarding diet, the participants expressed that they should increase the consumption of vegetables and fruits on a daily basis, as well as the consumption of protein through fish consumption. Most of the participants ate more meat and eggs than fish. Some examples of high school students’ statements during the activity:

*“I should eat more fruit and vegetables”;*

*“The recommendations say 5 fruits a day and sometimes I don’t eat any”;*

*“I have never liked fish but I recognize that to have a healthy diet I should eat more”.*

They also stated the need to decrease the consumption of fast food, high-sugar food, and sugary and caffeinated drinks:

*“I admit that I should eat less fast food”;*

*“Eat less industrial pastries”;*

*“Less drinks like coke or fizzy drinks”.*

In general, the participants perceived a good practice of physical activity. However, some of them found that they should improve it, identifying less use of public and private transport, as well as less use of elevators as facilitators of a more active lifestyle:

*“I practice football two times a week but I could do more”.*

*“For example going to school cycling or on foot, instead of taking the bus”.*

*“We are used to use the elevators and we should take more stairs”.*

High school students considered that they should improve their resting time, proposing to decrease cell phone use before going to sleep. The majority agreed that they use the cell phone too many hours a day, during bedtime and just before falling asleep:

*“I have to leave my cell phone before I go to sleep”.*

Emotional health was another area identified by high school students to be improved. At this point, participants stated different needs. Firstly, most of them identified the need to enhance self-confidence. Secondly, they identified the need to improve the ability to manage some emotions, such as fear and anger. Thirdly, some of the participants pointed out they had to appreciate more the positive things that happened to them. Finally, some of them stated that they should avoid becoming frustrated:

*“Not to be so dependent on what others tell about me, have more self-esteem”;*

*“I have to learn to handle fear and anger”;*

*“I get frustrated very easily and I need to improve it”.*

The last area the students identified as a bad habit for a healthy life was tobacco consumption. Although during the session, some high school students stated that they used tobacco, no intention of changing their habits was pointed out through the mind map. 

### 3.2. Results Related to the Nursing Students

Twelve students from the first year of the nursing degree participated in the study. Of those, 91% identified as female, and the average age was 19 (*SD = 1.2)*. Through their reflective journals, we observed the results related to their participation in a service learning activity.

All the participants agreed with a positive assessment of the activity on health habits using service learning:

*“I think it is a pretty good proposal for students in their first year at the university”;*

*“I didn’t imagine it would be like this, and I value it very positively”.*

The students stated that participating in the project allowed them to learn effectively about health habits, especially during the preparation of the session. Moreover, through their reflections and the research process, they could identify the possible queries that might arise during the sessions:

*“I didn’t imagine it would be like this, and I value it very positively”;*

*“We had to look up much information to prepare the session and this allowed us to learn”;*

*“While we were building the presentation we considered some of the doubts that could arise during the sessions. This made us reflect on the topic and look for more information if we felt it was necessary”.*

Most of the participants pointed out that by service learning, they should learn other topics closely related to the nursing profession:

*“I think it’s a very good way to learn all the topics presented in this subject, but I suppose it should be very difficult to organize…”.*

They also manifested that participating in the project helped them to improve their communication skills and their confidence in public speaking:

*“Also, I value very positively the fact of making the presentation. I was a little shy and sometimes I get nervous when I have to talk in public. For the session I practiced a lot, and when I did it I felt I was capable of it. This has improved my confidence as well as my communication skills”.*

The students of the Nursing Degree rated the experience of carrying out a nursing activity in the community in a real environment during the first year of university training very positively. They also reported that it is a good input for them to know about the activities developed in community care:

*“In my opinion, it is very important to get involved in real activities such as these because we will do it in the future, as nurses”;*

*“For me, making a session as a nurse was exciting. Also, knowing the role of community nurses with teenagers”.*

Finally, most nursing students expressed feeling prepared and comfortable while participating in the project:

*“I think the high school students were happy and learned about health habits with our session”;*

*“We worked very hard preparing the session and I think afterwards we were ready to do it“.*

Finally, all the nursing students who participated in this activity completed the six stages encompassed in the Gibbs Reflective Cycle [23] in their reflective journals. 

## 4. Discussion

The learning service requires a network that promotes and guarantees the articulation of formal educational institutions with the social organizations that intervene in the reality intending to influence through the development of the service [11]. For this study, a network between the university, the high school, and the community healthcare setting of the territory was established. The study results showed that promoting healthy habits through an activity of service learning as a teaching methodology provides benefits for nursing students and also for high school students. 

### 4.1. High School Students

Service learning has a social utility [11]. By providing experiences that are linked with conscious educational growth and emphasizing the accomplishment of tasks that meet human needs, service learning activities address social problems [29]. 

Several studies showed that positive lifestyle choices and behaviors are the foundation of good health [30,31,32], and in this sense, the results of the present study indicate that high school students who participated in the activity could identify some health habits that needed to be improved, such as diet, physical activity, rest, and emotional health.

Regarding diet, high school participants agreed on the need to increase the consumption of daily vegetables, fruits, and fish and decrease the consumption of fast food, high-sugar food, and sugary and caffeinated drinks. The results are in line with World Health Organization recommendations for a healthy diet in teenagers [33]. According to Yusuf and Fleming, diet in adolescence has a great transcendence in the projection of the quality of adult life. Dietary habits established during this stage of life are likely to persist into adulthood; in addition, consuming a healthy diet as a teenager helps to protect against malnutrition and non-communicable diseases [34,35]. 

Physical inactivity is associated with many non-communicable diseases and substantial economic costs on a global scale [36]. In order to deal with these situations, many strategies are being developed, such as the Global Plan on Physical Activity 2018–2030 [37]. Findings from this study showed that the teenagers detected they should be more active, which is in line with global surveillance data observed that approximately 20% of under-18 s claimed to be sufficiently active [38].

Tarokh [39] supported the important role of resting time and sleeping habits in teenagers as they have a huge impact on brain function and behavior establishment. Moreover, Carskadon [40] suggested nine tips to help teenagers to improve their sleeping habits. Some of them are as follows: avoid caffeine after school, avoid “arousing” activities in the evening, and do not fall asleep with your cell phone. In accordance with our results, it was shown that teenagers proposed to decrease cell phone use before bedtime in order to improve their rest. 

Regarding the need to improve emotional health habits, the study results indicated that teenagers should enhance their self-confidence and abilities to manage fear, anger, and frustration. A recent systematic review conducted by Amorós addressed more emotional problems, anxiety, depression, and stress in Spanish children and teenagers due to the COVID-19′s lockdown [41]. Therefore, our findings could be explained by the moment when the data were collected. 

According to WHO, the tobacco epidemic is one of the biggest public health threats the world has ever faced, and its use often begins in early adolescence [1]. The initiation and establishment of smoking behavior frequently occur during adolescence, and 9 of 10 children started smoking by the age of 18 years old [42], with 10% of 13–15-year-olds being worldwide smokers [1]. In addition, the prevalence of tobacco consumption among teenagers retrieved from the Global Health Observatory was 19,33%, especially in high-income countries [43]. Although some high school students in this study stated that they consume tobacco, they did not express any intention to change this habit. Therefore, in our territory, strategies are necessary to raise awareness about the harmful effect of tobacco on health among adolescents. In this line, a Health and School program could be a good scenario to promote these strategies. School health programs have been demonstrated to be the most cost-effective way to influence health behaviors in young people [44]. 

### 4.2. Nursing students

Previous studies have shown service learning benefits to those putting it into practice in terms of learning content, skills, and values [11]. Students learn through the application process of integrating theoretical concepts, problem-based learning, and reflective practice [15,16,17], which according to Evans, favors learning outcomes [45].

Participating in an activity of service learning increases the perception of knowledge about healthy lifestyles among nursing students. Using this methodology is considered to be positive, not only to improve students’ nursing health but also to impact the community’s health because, as future nurses, they will play a leading role in this task [46].

Communication is another skill that nursing students could increase while developing the activity of service learning in a high school. Previous studies have described communication as a crucial element in all nursing activities and interventions, such as prevention, treatment, rehabilitation, education, and health promotion [47].

It was also found in our study that carrying out a nursing activity in a real environment during the first year of university training is positive for nursing students learning, especially because they feel this work has a positive effect on teenagers. These results are confirmed by other research that found service learning as a good strategy to bridge the gap between theory and practice, while critical thinking and civic responsibility are promoted [14].

Nursing students also reported it being a good input for them to know about the activities developed in community care. According to Bulot and Johnson (year), participating in activities such as these is a mechanism to better understand and relate to social conditions from the perspective of the community in which they are engaged [48], which helps to develop more professional awareness [49].

### 4.3. Implication for Practice

According to Adegbola [50], in order to reach high standards in education, it is necessary to present activities crafted with the intention to induce learning while they are also applicable to real-life situations. Nursing lecturers can increase their effectiveness, including service learning and reflection on their topics, as well as engaging with civic-minded healthcare professionals. 

This study aimed to assess a service learning activity on health habits in high school teenagers developed for nursing students with the best knowledge of both. The results found with this activity could be a guide for nursing lecturers, other university educators, and faculty managers to develop similar activities promoting and motivating actions to encourage learning strategies in order to obtain better learning experiences for university students.

### 4.4. Limitations

There are some limitations to the findings of the present study. Firstly, the subject of Healthy Person throughout the Life Cycle developed in the first year of a nursing degree in our region may not be taught in other nursing faculties, which could make it more difficult to replicate the service learning experience. However, health habits are explained in some modules of the degree, so this activity could be adapted to other subjects. Another limitation is regarding qualitative research as the population of the study is only from one geographical region and with students with a similar socioeconomic background. This could limit the transferability of the results. Moreover, the fact that the nursing students who participated were those who had performed the best work and were more motivated by the service-learning activity could be another limitation.

## 5. Conclusions 

The study findings showed that the service learning activity on health habits conducted by nursing students in a high school has a positive effect on the knowledge of healthy habits for both participants, nursing and high school students. Participating in an activity of service learning improves communication skills among first-year nursing students and narrows the gap between university theory and nursing practice. 

## Data Availability

The data presented in this study are available on request from the corresponding author. Data are not publicly available due to the privacy term signed by the participants in the informed consent.
